# Detection of Gliadin‐Activated CD4 ^+^ T Cells Is a New Assay to Reveal Pathogenic Lymphocytes in Celiac Disease

**DOI:** 10.1111/jcmm.70898

**Published:** 2025-10-22

**Authors:** Laura Pisapia, Marcella D'Ambrosio, Ilaria Mottola, Stefania Picascia, Domenico De Girolamo, Fabiana Castiglione, Nadia Tinto, Antonio Rispo, Carmen Gianfrani, Giovanna Del Pozzo

**Affiliations:** ^1^ Institute of Genetics and Biophysics CNR Naples Italy; ^2^ Institute of Biochemistry and Cell Biology CNR Naples Italy; ^3^ Institute of Endotypes in Oncology, Metabolism and Immunology “G. Salvatore” CNR Naples Italy; ^4^ Department of Molecular Medicine and Medical Biotechnology CEINGE Advanced Biotechnologies “Franco Salvatore” Naples Italy; ^5^ Gastroenterology, Department of Clinical Medicine and Surgery University Federico II Naples Italy

**Keywords:** autoimmunity, CD4 lymphocyte activation markers, diagnosis, gliadin

## Abstract

The diagnosis of celiac disease (CD) relies on the presence of serum antibodies against type 2‐tissue transglutaminase (tTG2) and endomysium, which in adult patients must be confirmed by assessing small intestinal mucosa damage through esophagogastroduodenoscopy. We aim to establish a non‐invasive method focused on detecting activated CD4^+^ T cells that are reactive to gliadin in the peripheral blood of patients with untreated CD. Peripheral blood mononuclear cells from 20 patients with untreated CD, 17 patients on a gluten‐free diet and 10 healthy donors were stimulated in vitro with whole gliadin or immunodominant peptides. After 48 h, cells were stained with fluorochrome‐conjugated monoclonal antibodies against OX40 and 4‐1BB surface activation markers. Gliadin‐specific, activated CD4^+^ T cells were detected through multiparametric flow cytometry. OX40 and 4‐1BB activation markers are upregulated on CD4^+^ T cells following the engagement of the HLA‐DQ2.5‐gliadin complex with the T‐cell receptor (TCR). The frequency of gliadin‐specific, CD3^+^/CD4^+^/OX40^+^/4‐1BB^+^ cells is significantly higher in untreated than in treated patients and healthy controls and shows a positive correlation with the anti‐tTG2 antibody titers. This assay, defined as the *G.A.T.CD4* (*Gsliadin‐activated CD4* ^*+*^ *T cells*) method, might support CD diagnosis, particularly in doubtful cases having low autoantibody serum titers. *G.A.T.CD4* can be of help in monitoring disease progression, as the pathogenic T cells expressing OX40 and 4‐1BB activation markers are undetectable in the blood of treated patients. In the future, we aim to propose the *G.A.T.CD4* instead of esophagogastroduodenoscopy to perform accurate and less invasive diagnosis.

## 1 Introduction

Celiac disease (CD) is a chronic inflammatory disorder due to an abnormal immune reaction to gluten proteins. CD4^+^ T cells reactive to gluten have a key role in the inflammatory cascade that, in the most severe cases, leads to the destruction of the small intestinal mucosa architecture and functionality. Specifically, the ingestion of proline‐rich gluten proteins of wheat, rye and barley in patients affected by CD induces the production of antibodies to type 2‐tissue transglutaminase (tTG2) and to endomysium (EMA) in the serum and the activation of CD4^+^ T cells reactive toward gluten antigens post‐transcriptionally deamidated by tTG2 [1] According to the recent guidelines of the European Society of Paediatric Gastroenterology (ESPGHAN), the CD diagnosis in children is made in the presence of clear symptoms and anti‐tTG2‐positive serology without the invasive practice of the gastrointestinal endoscopic investigation (EGDS) to sample jejunal biopsy [2]. In adults, the diagnostic work‐up requires intestinal biopsies performed in those subjects with positive serology and clear symptoms [3]. Following the diagnosis, either adult or paediatric patients have to follow a strict gluten‐free diet (GFD) regimen, which to date represents the only efficacious therapy to obtain seroconversion, symptom remission and small intestinal damage recovery [4].

The great majority of CD patients carry the HLA genes encoding the DQ2 or DQ8 heterodimers that have a key role in presenting gluten epitopes to cognate CD4^+^ T cells [5]. The HLA‐DQ2/DQ8‐restricted gluten‐specific T cells have been detected in the intestinal mucosa and the blood of CD patients but not in healthy subjects [6], thus supporting the pivotal role of the adaptive immune response in CD pathogenesis [6]. The most sensitive and specific assay to reveal the gluten‐specific CD4^+^ T cells is based on HLA‐tetramer technology [7, 8]. This assay requires the knowledge of cognate peptides and their MHC restricting elements and is related to the cell activation status only if combined with CD38 marker expression [9, 10]. The conventional and more practical techniques used to detect gliadin‐specific CD4^+^ T cells in CD patients are based on the measurement of IL‐2 or IFNγ cytokines, produced following ex vivo antigen stimulation. Among these approaches, there are immune‐enzymatic assays, including sandwich ELISA, to quantify cytokines released in the culture supernatant [11] and the ELISPOT to measure the single cell cytokine‐secreting [12]. Other studies have demonstrated that an increase in serum IL‐2 is correlated with the timing and severity of symptoms in patients with active CD or in treated patients after a gluten oral challenge [13]. Recently, a whole blood interleukin IL‐2 release assay (WBAIL‐2) has been developed for the detection of gluten‐specific T cells and proposed as a diagnostic tool for CD, even in patients consuming a gluten‐free diet [13].

Overall, all these assays have a limitation in detecting rare cytokine‐producing T cells circulating in peripheral blood, as gliadin‐specific T cells are. Furthermore, due to the functional heterogeneity of the antigen‐responsive CD4^+^ T cells, the analysis of a single cytokine might significantly underestimate the magnitude of the antigen‐specific T cell‐mediated response [7, 8, 9, 10].

These limitations in revealing antigen‐responsive CD4^+^ T cells can be overcome by the activation‐induced marker (AIM) assay, which measures the upregulation of selected surface markers following the TCR stimulation by specific antigens. More specifically, the AIM assay is a highly sensitive and cytokine‐independent detection of Ag‐specific T cells [14, 15, 16]. The antigen‐specific T‐cell mediated responses play a fundamental role in autoimmune disorders (directed against self‐antigens) but also in natural infections and in protective vaccine strategies [17]. The AIM assay has been largely used to identify and monitor activated T lymphocytes in the peripheral blood of patients affected by COVID‐19 and in subjects vaccinated to assess the activation of the protective cellular immune response, thus allowing the identification and quantitation of SARS‐CoV‐2 specific CD4^+^ and CD8^+^ T‐cell responses [18, 19, 20]. Among several TCR‐dependent activation markers, we selected OX40 (CD134) and 4‐1BB (CD137), which are both members of the tumour necrosis factor receptor superfamily (TNFRSF) and expressed by CD4^+^ and CD8^+^ T cells. The OX40 molecule binding the OX40L ligand on APC is not expressed on unprimed T cells, but its expression increases after 24 h of cell stimulation. OX40 regulates the development of effector CD4^+^ T cells by sustaining clonal expansion and prolonging cell survival. Following the effector phase, OX40 also boosts the generation of memory CD4^+^ T cells and inhibits the development of Treg cells. The other activation marker, 4‐1BB, binds CD137L on APC, induces proliferation and cytokine production and enhances the survival of both CD4^+^ and CD8^+^ T cells. The 4‐1BB upregulation starts earlier than OX40, 6 h after TCR engagement by the cognate HLA‐peptide complex and, similarly to OX40, its upregulation is independent of the cytokine secretion and differentiation stage of CD4^+^ T cell [21].

In this study, we adapted the concept that TCR stimulation by the cognate gliadin‐HLA complex induces upregulation of activation surface markers to develop a novel method for CD diagnosis named *Gliadin‐activated CD4* ^+^ *T cells* assay (*G.A.T.CD4* patented method) that permits the identification of CD4^+^ T lymphocytes reactive toward the most dominant gliadin peptides [22]. Specifically, we detected the presence of gliadin‐specific CD4^+^ T lymphocytes by counting the OX40 (CD134) and 4‐1BB (CD137) double‐positive CD3^+^CD4^+^ cells in the peripheral blood of patients with untreated CD or treated CD patients on GFD and in healthy controls.

## 2 Material and Methods

### 2.1 Study Design and Cohort

The study population included 20 subjects with untreated CD enrolled at the time of diagnosis, 17 treated patients on at least 6 months of gluten‐free diet, and 10 healthy donors. All patients were adults (between 25 and 65 years old) carrying the HLA‐DQ2 genotype (Table S1). The inclusion criteria of patients with untreated CD were the presence of clinical symptoms, anti‐tTG2‐IgA antibodies > 7 U/mL or anti‐EMA‐IgA positive serology. All diagnoses of untreated CD were biopsy‐proven. Histological examination of duodenal biopsies showed the presence of villous damage with Marsh 2–3 grade lesions. The inclusion criteria of treated CD subjects were the absence of clinical symptoms, anti‐tTG2‐IgA antibodies < 7 U/mL and anti‐EMA‐IgA negative. Anti‐TG2 and anti‐EMA antibodies were measured by ELISA (kits provided by Delta Biologicals s.r.l., Rome, Italy). The exclusion criteria were: age under 18 years, inability or failure to give informed consent, doubtful or discordant CD‐antibody profile, lack of jejunal biopsy evaluation and/or clinical and serology data at diagnosis or follow‐up. Treated CD patients who declared poor compliance with the gluten‐free diet were excluded. The study was approved by the Ethical Committee of the University Federico II of Naples, Italy (protocol n°178/19). The IRB approval date was 12 December 2019, and the amendment dated 3 December 2020, extended the project to 6 December 2023. Written informed consent for participation in the study was obtained from each patient before the start of the study. The study was conducted according to the Good Clinical Practice guidelines and the Declaration of Helsinki.

### 2.2 Gliadin Activated CD4 ^+^ T Cells (*G.A.T.CD4*) Method

Peripheral blood mononuclear cells (PBMCs) were isolated from 10 mL blood samples by Ficoll‐Paque PLUS gradient separation (GE Healthcare BioScience). An aliquot of recovered PBMCs (2 × 10^6^) was used for DNA extraction by FlexiGene DNA kit (QIAGEN) to carry out the HLA typing. HLA‐DQ genotyping was performed using Sequence Specific Primers (SSP)‐PCR, employing Histo Type Celiac Disease and DQB low kits (Astra Formedic s.r.l.). The remaining PBMCs were cryopreserved until functional experiments were conducted. PBMCs (1 × 10^6^/96‐well plate) were cultured in 200 μL of RPMI supplemented with 10% FBS and antibiotics and pre‐treated with 0.5 μg/mL of anti‐CD40 monoclonal antibody (Miltenyi Biotech) for 15 min at 37°C. Cells were then stimulated for 48 h with a pool of five highly immunogenic peptides, indicated as Pool 1–5, including epitopes belonging to the gliadins α‐[DQ2.5‐glia‐α1a/α2], ω‐[DQ2.5‐glia‐ω1/2] and γ‐[DQ2.5‐glia‐γ1, DQ2.5‐glia‐γ2, and DQ2.5 γ‐glia 26 mer] (see Table S2) at a final concentration of 10 μg/mL (2 μg/mL for each peptide). In parallel, cells were also stimulated with a deamidated pepsin‐trypsin digest of whole gliadin, indicated as PT‐G, at 50 μg/mL. As positive control cells were stimulated with the phytohemagglutinin (PHA, Invitrogen‐ThermoFisher) at 2 μg/mL, while the culture medium served as the negative control. Forty‐eight hours after antigen stimulation, the PBMCs were harvested, washed, resuspended for flow cytometry analysis in ice‐cold FACS buffer (PBS plus 0.5% FBS) and incubated at 4°C for 30 min with fluoresceinated mAbs anti‐CD3‐FITC, anti‐CD4‐PE, anti‐OX40‐PECy7 and anti‐4‐1BB‐APC (Sony Biotechnology, distributed by Cytosens, Italy). After incubation, cells were washed twice with FACS Buffer before flow cytometer analysis. The immunophenotyping was analysed using the BD FACSCanto II, and the data were processed using the BD FACSDiva Software (BD Biosciences). A representative gating strategy is shown in Figure S1. Live lymphocytes were distinguished from other cells in whole PBMCs sample by their uniform physical properties, characterised by intermediate forward scatter (FSC‐A) and low side scatter (SSC‐A).

Activated, gliadin‐specific T cells were counted within the CD3^+^/CD4^+^ gated population and identified as 0X40^+^/4‐1BB^+^ double‐positive cells [21]. The number of activated cells is expressed as the percentage of CD3^+^/CD4^+^/OX40^+^/4‐1BB^+^ cells in response to gliadin antigen stimulation.

### 2.3 Measurement of IFNγ Production by ELISPOT

PBMCs collected from CD patients and healthy donors were cultured in the same conditions used in the *G.A.T.CD4* assay. Cells were plated at 3 × 10^5^ in 200 μL on 96‐well nitrocellulose‐backed plates (MAHAS4510; Millipore) coated with 5 μg/mL of primary anti–IFNγ mAb (Mabtech) and incubated for 40 h at 37°C in the presence of Pool 1–5, PT‐G and PHA at the same concentrations used for the *G.A.T.CD4* assay. Unstimulated cells cultured in medium alone were included as a negative control. After incubation, plates were extensively washed with PBS/0.05% Tween‐20 and incubated with 2 μg/mL of secondary anti‐IFNγ biotinylated mAb for 2 h followed by a 1‐h incubation of streptavidin‐HRP (BD Pharmingen). Spots were developed by adding aminoethyl carbazole (Sigma‐Aldrich) solution and counted using an ImmunoSpot image analyser (A.EL.VIS). Data are expressed as IFNγ‐Spot‐Forming Cells (IFNγ‐SFC) per 10^6^ PBMCs. Each experimental point was plated in duplicate.

### 2.4 Statistical Analysis

All statistical analyses were performed using GraphPad Prism 8.0. The Student *t*‐test was used either paired or unpaired, with the two‐tailed distribution depending on the type of comparison. Before performing the Student *t*‐test, we assessed the normality of the distribution of each group's data using the Shapiro–Wilk test. Statistical analysis was also performed by ANOVA test (data not shown). Sensitivity and specificity of the data were confirmed by the ROC analysis (Figure S3). Correlations between anti‐tTG2 antibody titers and the percentages of activated gliadin‐specific CD4^+^ T cells were evaluated using *Pearson's* correlation test.

## 3 Results

### 3.1 Gliadin‐Activated CD4 ^+^ T Cell Assay (*G.A.T.CD4*) Detects CD‐Specific T Cells in Untreated Patients

**FIGURE 1:**
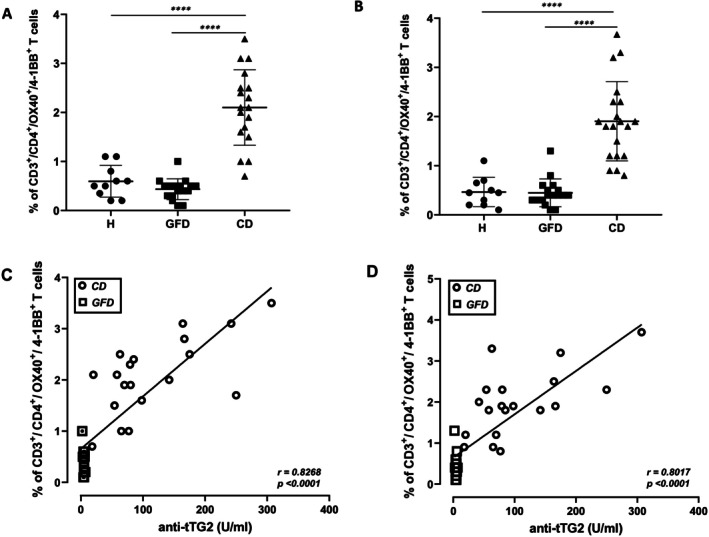
CD4^+^ T‐cell response to gliadin stimulation measured in celiac patients by *G.A.T.CD4* assay and correlation with titers of anti‐tTG2 antibodies. The figure shows the percentages of CD3^+^/CD4^+^/OX40^+^/4‐1BB^+^ cells after stimulation of PBMCs with gliadin peptides Pool 1–5 (Panel A) or with an enzymatic digest of gliadin (PT‐G) (Panel C) detected in healthy donors (H), treated celiac patients (GFD) and untreated patients (CD). The lines representing the mean values are indicated. The unpaired Student *t*‐test was applied for statistical significance. ****p < 0.0001. The significance of correlation between the serum anti‐tTG2 titers and the percentage of CD3^+^/CD4^+^/OX40^+^/4‐1BB^+^ T cells upon stimulation with gliadin peptides, Pool 1–5 (Panel C) and deamidated gliadin, PT‐G (Panel D) was calculated by the *Pearson* test. The value of correlation coefficient *r* is indicated.

Immune responsiveness to gliadin antigens was assessed in PBMCs from adult celiac patients and healthy non‐CD donors. The percentages of circulating T cells specific for gliadin antigens were measured within the subset of CD3^+^CD4^+^ cells expressing the early activation OX40 and 4‐1BB surface markers. Figure 1 shows the percentages of CD3^+^/CD4^+^/OX40^+^/4‐1BB^+^ cells detected in healthy donors (H), treated patients on GFD (GFD) and untreated patients (CD) after stimulation with Pool 1–5 (Panel A) and with PT‐G (Panel B). The highest frequencies of activated CD4^+^ T cells were detected in patients with untreated CD, that is, at the time of disease diagnosis, for both antigenic conditions. More specifically, the percentages of CD3^+^/CD4^+^/OX40^+^/4‐1BB^+^ cells in response to Pool 1–5 were (mean value and range): 0.8% (0.2–1.1) in healthy donors, 0.4% (0.1–1) in GFD patients and 2.10% (1–3.5) in untreated CD patients (Figure 1, Panel A). A similar number of responder cells was observed in response to PT‐G stimulation (mean value and range): 0.47% (0.1–1.1) in healthy subjects, 0.4% (0.1–1.3) in GFD patients, and 1.9% (0.8–3.7) in untreated CD patients (Figure 1, Panel B). The percentage of positive cells measured in the absence of antigen (medium alone), as well as a dot plot of CD3^+^/CD4^+^/OX40^+^/4‐1BB^+^ cell population for a representative subject from the three cohorts analysed, are reported in Figure S2.

Our findings demonstrate that the upregulation of OX40 and 4‐1BB surface markers occurred in the gliadin‐specific CD4^+^ T cell population only in patients with untreated disease. In fact, the frequency of activated cells measured in gliadin/peptide‐stimulated cultures of PBMCs of treated CD patients is comparable to that found in non‐celiac donors. To assess the sensitivity and specificity of our test, we performed a ROC analysis comparing the percentages of CD3^+^/CD4^+^/OX40^+^/4‐1BB^+^ cells from untreated CD patients versus treated GFD patients, after gliadin antigens stimulation. ROC analysis provided the optimal cut‐offs for all CD patients compared with all GFD patients after Pool 1–5 stimulation (cut‐off: 0.7; Figure S3, Panel A), yielding an area under the ROC curve (AUC) of 0.9938 (95% confidence interval [CI]: 0.97–1.00), and 0.9809 (95% CI: 0.94–1.00) for data obtained with PT‐G stimulation (cut‐off: 0.9; Figure S3, Panel B).

The corresponding sensitivity and specificity for untreated CD versus GFD were respectively 0.95 (95% CI: 0.86–0.99) and 0.94 (95% CI: 0.86–0.99) for Pool 1–5 stimulation, and 0.94 (95% CI: 0.86–0.99) and 0.94 (95% CI: 0.8–0.99) for PT‐G stimulation.

Moreover, a direct and significant correlation was observed between the serum titers of anti‐tTG2 antibodies from all untreated CD and GFD patients and the percentages of activated CD3^+^/CD4^+^/OX40^+^/4‐1BB^+^ cells in response to both Pool 1–5 (Figure 1, Panel C) and PT‐G (Figure 1, Panel D) stimulations.

**FIGURE 2:**
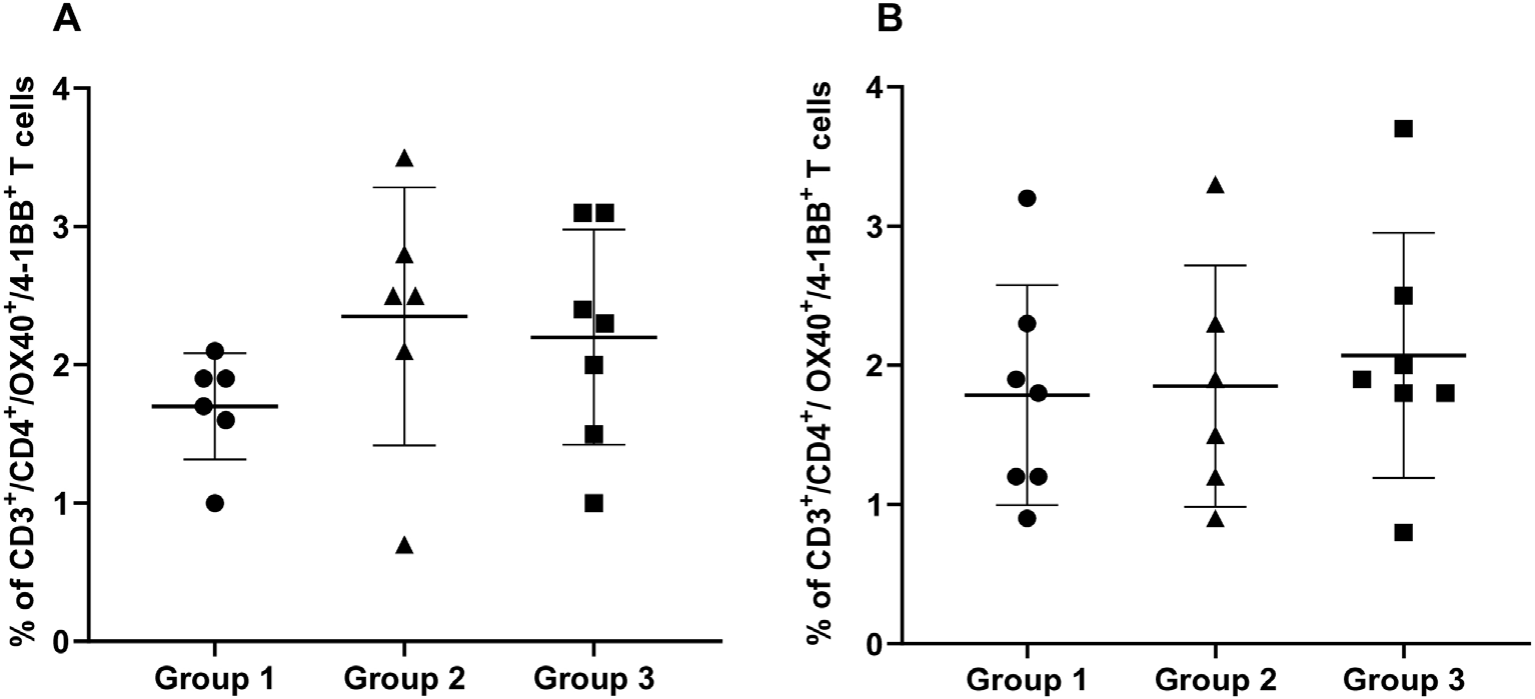
Frequencies of activated gliadin‐specific CD4^+^ T cells in relation to patients' HLA‐DQ genotypes. The percentages of CD3^+^/CD4^+^/OX40^+^/4‐1BB^+^ cells measured in PBMCs stimulated with Pool 1–5 (Panel A) and PT‐G (Panel B) of patients with active disease were shown according to their genotype. Group 1: DR3/DR3 or DR3/DR7 patients; Group 2: DR3/DR5 and DR5/DR7 patients; Group 3: DR1/3 and DR3/DRX patients (see Table S1). The differences among the groups are not significant.

Then, we analysed the frequency of CD3^+^/CD4^+^/OX40^+^/4‐1BB^+^ cells detected in untreated patients in relation to their HLA genotype. The subjects carrying the DR3/DR3 or DR3/DR7 genotypes, expected to express 100% of DQ2.5/DQ2.2 heterodimers, represent group 1. Group 2 includes patients carrying the DR3/DR5 or DR5/DR7 genotypes, assumed to display 50% of DQ2.5/DQ2.2 heterodimers on their cells, and finally, group 3 comprises patients with DR1/DR3 or DR3/DRX genotypes, expressing 25% of DQ2.5/DQ2.2 molecules [23]. As shown in Figure 2, no significant differences in the percentage of CD3^+^/CD4^+^/OX40^+^/4‐1BB^+^ cells were observed among the patients in response to both gliadin immunodominant peptides (Panel A) and whole gliadin (Panel B), thus indicating a similar capability of *G.A.T.CD4* method to detect activated gliadin‐specific CD4^+^ T cells in CD patients despite the HLA‐DQ2 genotypes. We also carried out experiments with PBMCs from two treated and two untreated heterozygous DQ8 patients. Similarly to HLA‐DQ2 patients, we found the presence of CD3^+^/CD4^+^/OX40^+^/4‐1BB^+^ cells in the PT‐G stimulated PBMCs from patients with active disease (data not shown).

### 3.2 Comparison Between Different Assays in Response to Gliadin‐Antigen Stimulation

**FIGURE 3:**
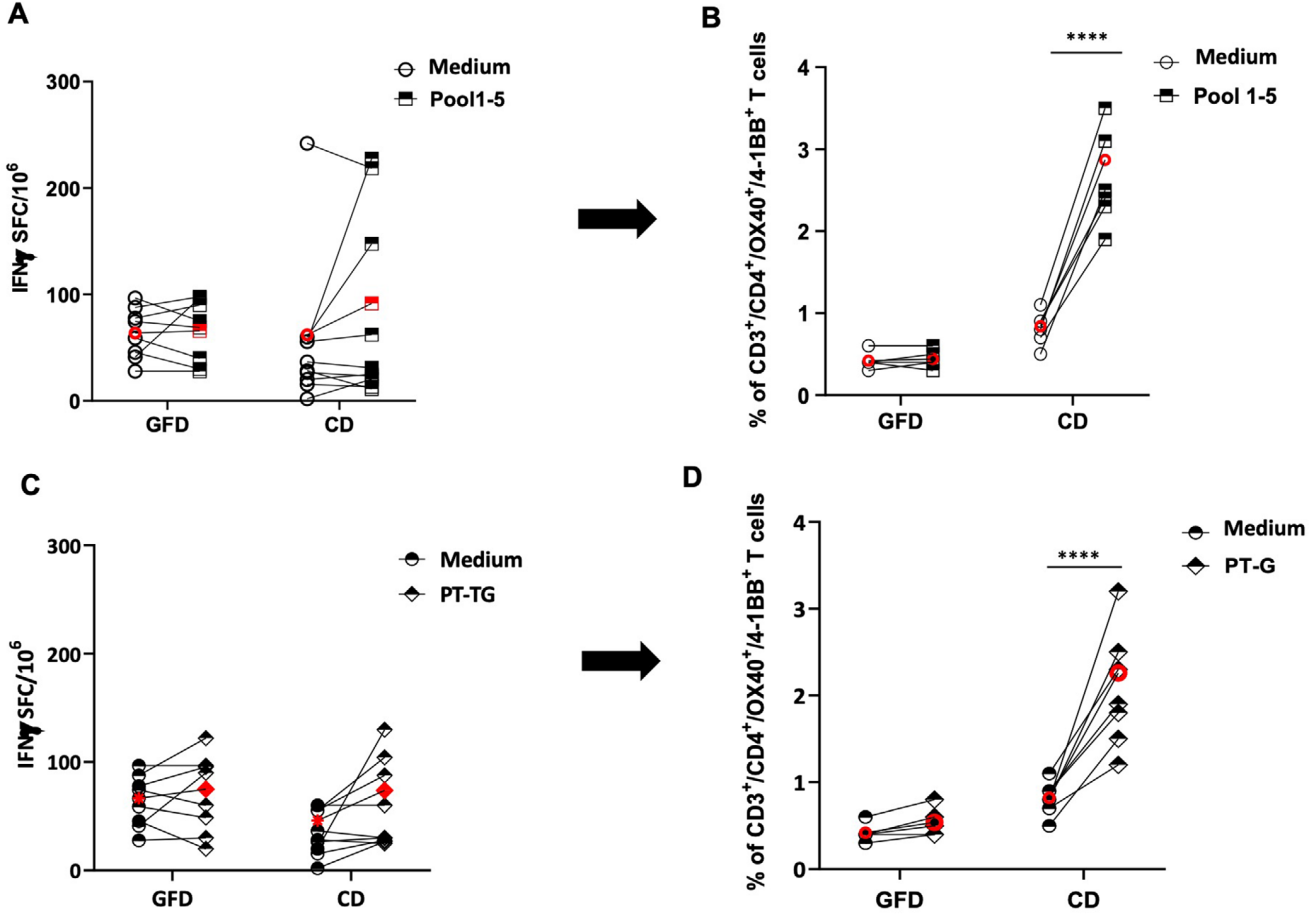
Comparative detection of gliadin‐specific CD4^+^ T cells assessed by *G.A.T.CD4* and by IFNγ ELISPOT assays. The anti‐gliadin CD4^+^ T‐cell responses were assayed in the PBMCs by IFNγ‐ELISPOT upon Pool 1–5 (Panel A) or PT‐G (Panel C) stimulation, with respect to proper baseline (Medium), in 8 GFD patients and 10 patients with untreated CD. The IFNγ‐secreting T cells are reported as spot‐forming cells (SFC)/10^6^ PBMCs. Panels B and D reported the percentages of CD3^+^/CD4^+^/OX40^+^/4‐1BB^+^ cells detected in five patients on GFD and 7 untreated CD patients activated by Pool 1–5 (Panel B) or PT‐G (Panel D) stimulations. Results of *G.A.T.CD4* assays have already been included in Figure 1 and are reported for comparative purposes. The mean values are indicated in red. The paired Student *t*‐test was applied for statistical significance. ****p < 0.0001.

ELISPOT is a widely used assay to detect antigen‐specific T cells producing IFNγ. PBMCs were stimulated with gliadin/peptides under the same conditions as the *G.A.T.CD4* test and assayed for IFNγ production by ELISPOT. More specifically, based on cell availability, we stimulated the PBMCs from 8 GFD‐treated patients (CD154, CD163, CD165, CD170, CD171, CD178, CD180, CD194) and 10 untreated CD patients (CD85, CD159, CD173, CD174, CD176, CD179, CD184, CD181, CD183, CD192) previously assayed by *G.A.T.CD4* with both antigens. Furthermore, PBMCs from an additional three GFD‐treated (CD154, CD171, CD194) and three untreated patients (CD176, CD181, CD183) were included but not reported in Table S1. As shown in Figure 3, Panel A, no significant increase in IFNγ‐secreting cells was found in both groups of patients in response to either PT‐G and peptides. More specifically, the mean values of IFNγ‐secreting cells in response to Pool 1–5 (Panel A) were 65.7 SFC/10^6^ (range 27.7–97.7) in GFD patients and 91.5 IFNγ‐SFC/10^6^ (range 11.1–227.5) in untreated patients. Similarly, in response to PT‐G, the mean number of IFNγ‐SFC (Panel C) was 70.3 (range 19.9–122.1) for GFD patients and 128.7 (range 24.9–666.0) for untreated CD patients. The IFNγ‐SFC/10^6^ measured in unstimulated cells (medium) from GFD and untreated patients were 63.7 and 61.9, respectively. To compare the capability of the ELISPOT and *G.A.T.CD4* assays to detect gliadin‐specific T cells, we reported in Panels B and C of Figure 3 the frequencies of CD3^+^/CD4^+^/OX40^+^/4‐1BB^+^ cells (previously shown in Figure 1 and Figure S2) found in 5 GFD‐treated patients (CD163, CD165, CD170, CD178, CD180) and 8 untreated patients (CD85, CD159, CD173, CD174, CD179, CD183, CD184, CD192). In untreated CD, the frequency of CD3^+^/CD4^+^/OX40^+^/4‐1BB^+^ cells was 2.9% (range 1.9–3.5, Panel B) and 2.3% (range 1.2–3.2, Panel D) in response to Pool 1–5 and PT‐G stimulations, respectively, compared to a very low number of activated cells found in the unstimulated PBMCs, with mean values of 0.4 and 0.8, respectively, for GFD and untreated CD patients.

These comparative results demonstrate that the *G.A.T.CD4* test provides higher advantages with respect to the IFNγ‐ELISPOT in detecting gliadin‐reactive CD4^+^ T cells in subjects with a CD diagnosis.

## 4 Discussion

Assessment of the antigen specificity of CD4^+^ T cells is crucial for understanding the pathogenesis of autoimmune diseases. However, the identification of antigen‐responsive, pathogenic CD4^+^ T cells is complicated by their low frequency and functional heterogeneity. Multiple methodologies have been described to investigate CD4^+^ T cells ex vivo or after an in vitro expansion, in both peripheral blood and target tissue compartments, most of them based on cytokine measurements. In addition, since CD4^+^ T cells are a heterogeneous population and produce a large panel of cytokines/chemokines, the detection of antigen‐specific activated cells might underestimate the magnitude and breadth of the response.

In this study, we provided a new method defined as “*Gliadin‐activated CD4* ^+^ *T* cells, *G.A.T.CD4* ”, relied on the upregulation of activation markers on T lymphocytes upon a brief in vitro stimulation with gliadin antigen in subjects affected by celiac disease. Activation markers are surface proteins upregulated shortly after TCR engagement by antigen–HLA molecule complex. Their expression is independent of the cytokine‐secretion profile or cell differentiation stage [24]. Among several TCR‐dependent activation markers, we selected OX40 (CD134) and 4‐1BB (CD137), which play major roles as costimulatory receptors for CD4^+^ as well as CD8^+^ T cells and show an upregulation starting 24 h after antigenic stimulation of the TCR by HLA–peptide complex, reaching the expression peak after 48 h [14, 15, 16, 18]. This assay has been very useful in measuring the response of either CD4^+^ or CD8^+^ T cell populations against the many variants of concern of the Sars‐CoV‐2 virus [20, 25]. These studies took advantage of the use of large pools of overlapping peptides of spike and other main antigenic proteins of Sars‐CoV‐2 to stimulate human PBMCs, independently of knowledge of specific HLA Class I and II genotypes of analysed subjects [20, 25].

Celiac disease is a unique immune‐mediated condition in which both the antigenic determinants and the HLA restriction molecules of pathogenic CD4^+^ T cells are well known [6, 7, 12]. The *G.A.T.CD4* method has great diagnostic potential and should be used to avoid biopsy in adult patients. Technically, to adapt the assay to clinical use, we developed a protocol based on 5–6 × 10^6^ PBMCs, prepared from 10 mL of venom blood after a density gradient. As the PBMCs are frozen in liquid nitrogen before functional use, this allows us to collect a large number of samples before the assay. We used a pool of 5 immunodominant gliadin peptides and a tryptic‐peptic digest of gliadin protein to stimulate PBMCs of adult celiac patients enrolled at the time of disease diagnosis. After 2 days of incubation, the frequency of circulating CD4^+^ T cells expressing OX40/4‐1BB was calculated by cell staining with specific monoclonal antibodies and multiparametric flow cytometry analysis.

We found, in untreated CD patients, a frequency of positive cells with a mean value of 2.1%–1.9%, significantly higher than the percentage of these cells in treated patients, with a mean value of 0.4%, similar to that obtained in the healthy donors. These results indicated higher percentages of activated gliadin‐specific effector CD4^+^ T lymphocytes in the blood of patients with villous atrophy than in treated patients, whose frequency was similar to the healthy donors [7, 26, 27]. Most importantly, the anti‐tTG2 antibody serum titers found in untreated CD patients positively correlated with the frequency of gliadin‐activated CD4^+^ T cells. The rise of lymphocytes involved in the pathogenesis of the disease, in agreement with the antibody titer increment, supports our results and the potentiality of our assay in the diagnosis of celiac disease.

We have considered if the frequency of gliadin‐specific activated CD4^+^ T cells might vary based on the genotype of patients and analysed the results by dividing the participants into three groups according to their homozygous and heterozygous HLA‐DQ genotypes. We observed a comparable percentage of gliadin‐specific CD3^+^/CD4^+^/OX40^+^/4‐1BB^+^ activated cells, not dependent on the patient HLA haplotype. This conclusion is based on previous findings [26, 27] by our research team, demonstrating that the strength of the pathogenic autoimmune response by gliadin‐specific, memory CD4^+^ T cells is not strictly related to the HLA‐DQ genotype of APC but to the amount of gliadin antigen presented to the cognate cells. Indeed, two CD risk alleles DQA1*05 and DQB1*02 are more expressed than non‐CD‐associated alleles, causing a similar density of HLA‐DQ heterodimer by homozygous and heterozygous APC that present a similar number of HLA–gliadin complexes to responder T cells.

Finally, to evaluate the sensitivity of the method, we compared *G.A.T.CD4* with the IFNγ‐ELISPOT assay, a largely used method to analyse T‐cell mediated response in both basic and clinical research, in order to detect the gliadin‐specific CD4^+^ T cells. We observed a significant increase upon gliadin stimulation of CD3^+^/CD4^+^/OX40^+^/4‐1BB^+^ cells in all patients analysed by *G.A.T.CD4*, in contrast to a low frequency of IFNγ‐secreting cells that were detected only in a subgroup of tested patients by ELISPOT. Despite a large inter‐individual variability in the IFN‐γ production, with high background values in certain subjects, these comparative results do not affect the validity of IFNγ‐ELISPOT to monitor and enumerate gliadin‐reactive T cells in CD but indicate that the *G.A.T.CD4* test is more efficacious in revealing the presence of pathogenic T cells in the peripheral blood of patients with untreated CD. In addition, ELISPOT has never been used as a diagnostic tool but rather for research purposes, most likely because of the low specificity of the method applied to PBMC, since IFNγ is produced not only by CD4^+^ T cells but also by cytotoxic CD8^+^ T lymphocytes and TCRγδ^+^ lymphocytes.

Many studies have been published regarding the identification of activated gliadin‐specific CD4^+^ T cells in the blood of treated or untreated patients, mostly after a gluten oral challenge, through the HLA tetramers technology. These studies aimed to deepen the whole cell surface phenotype or transcriptomic profiles of gliadin‐specific CD4^+^ T cells with research, rather than diagnostic purposes [7, 28]. Moreover, in our study we confirmed the gliadin specificity of activated CD4^+^ T cells by *G.A.T.CD4* using two different antigen formulations and different statistical analyses of data.

In summary, we proposed the *G.A.T.CD4* method to detect and quantify the gliadin‐specific CD4^+^ T cells by monitoring the expression of surface activation markers OX40 and 4‐1BB. Further studies are in progress to make *G.A.T.CD4* a very efficacious diagnostic method in place of the esophagogastroduodenoscopy biopsy which, to date, represents the gold diagnostic standard for adult subjects. *G.A.T.CD4* is also a promising assay useful for monitoring disease remission after a gluten‐free diet.

## Author Contributions

**Laura Pisapia:** conceptualization (equal), formal analysis (equal), software (equal). **Marcella D'Ambrosio:** investigation (equal), methodology (equal). **Ilaria Mottola:** investigation (equal), methodology (equal). **Stefania Picascia:** methodology (equal), validation (equal). **Domenico De Girolamo:** methodology (equal), software (equal). **Fabiana Castiglione:** data curation (equal), formal analysis (equal). **Nadia Tinto:** data curation (equal), formal analysis (equal). **Antonio Rispo:** data curation (equal), resources (equal). **Carmen Gianfrani:** funding acquisition (equal), supervision (equal), writing – original draft (equal), writing – review and editing (equal). **Giovanna Del Pozzo:** conceptualization (equal), funding acquisition (equal), supervision (equal), writing – original draft (equal), writing – review and editing (equal).

## Consent

Informed consent was obtained from all subjects involved in the study.

## Conflicts of Interest

The authors declare no conflicts of interest.

## Supporting information


**Figure S1:** Gating strategy of gliadin‐specific, activated CD4^+^ T cells.
**Figure S2:** Gliadin‐activated CD3^+^/CD4^+^/OX40^+^/4‐1BB^+^ cells detected by *G.A.T.CD4* assay.
**Figure S3:** ROC analysis.
**Table S1:** Patients enrolled for *G.A.T.CD4* analysis.
**Table S2:** Immunodominant gliadin peptides included in Pool 1–5.
