# Supplementary material for: Detection of Gliadin‐Activated CD4 + T Cells Is a New Assay to Reveal Pathogenic Lymphocytes in Celiac Disease
Source: J Cell Mol Med. 2025 Oct 22;29(20):e70898. doi: 10.1111/jcmm.70898 (PMC12541239; doi:10.1111/jcmm.70898)
Supplement: Supplementary file 1 — Figure S1: Gating strategy of gliadin‐specific, activated CD4+ T cells. Figure S2: Gliadin‐activated CD3+/CD4+/OX40+/4‐1BB+ cells detected by G.A.T.CD4 assay. Figure S3: ROC analysis. Table S1: Patients enrolled for G.A.T.CD4 analysis. Table S2: Immunodominant gliadin peptides included in Pool 1–5. [file JCMM-29-e70898-s001.docx]

**
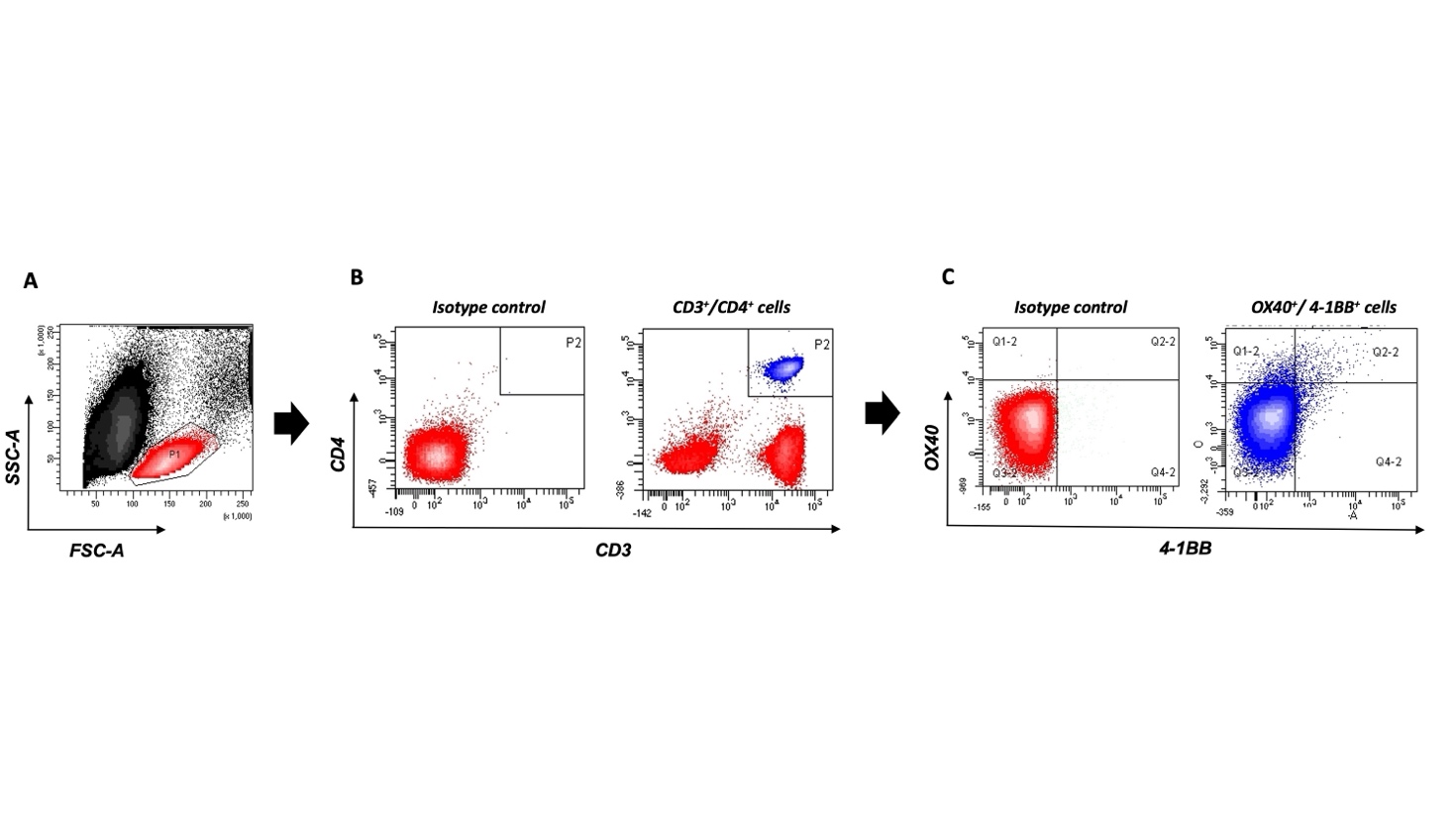
Supplementary Figure 1.** *Gating strategy of gliadin-specific, activated CD4^+^ T cells*

Live lymphocytes are selected based on size (FSC-A) and granularity (SSC-A) (**Panel A**), and selection of CD3^+^ and CD4^+^ surface expression (**Panel B**). Cell activation is measured as a percentage of OX40/4-1BB double-positive cells within the CD4^+^ T cell subset (**Panel C**).

**
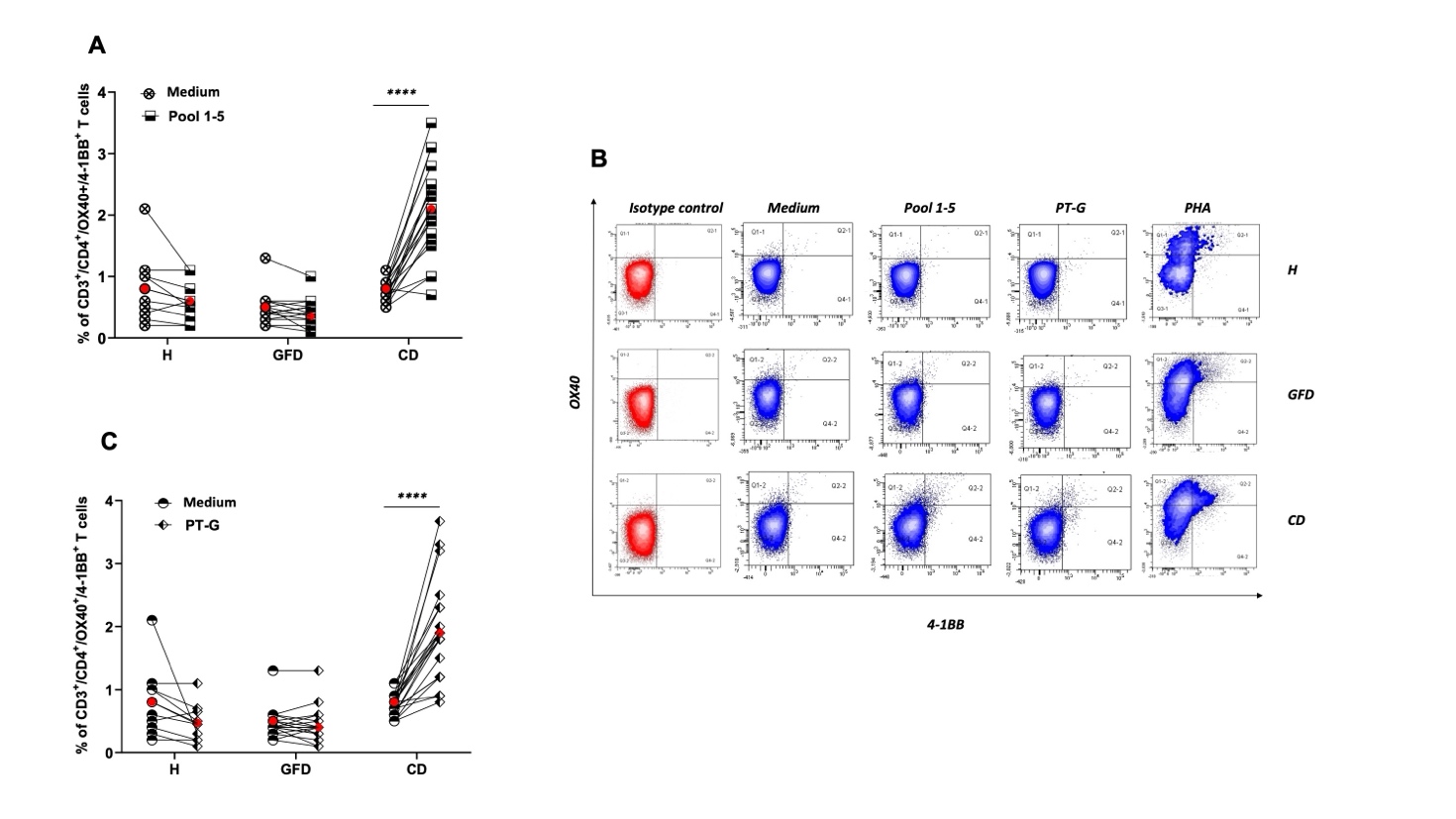
**

**Supplementary Figure 2.** G*liadin-activated CD3^+^/CD4^+^/OX40^+^/4-1BB^+^ cells detected by G.A.T.CD4 assay*

The figure shows the percentages of CD3^+^/CD4^+^/OX40^+^/4-1BB^+^ cells after stimulation of PBMCs with Pool 1-5 (**Panel A**) or with PT-G (**Panel C**) detected in healthy donors (H), treated celiac patients (GFD) and untreated patients (CD) compared to unstimulated cells (Medium), measured in all subjects analysed. The mean values are indicated in red. The paired Student’s *t*-test was employed to assess statistical significance. *****p* < 0.0001. **Panel B** shows representative flow cytometry dot plots of CD3^+^/CD4^+^/OX40^+^/4-1BB^+^ cells for one subject per group (H, GFD, CD). From left to right, the plots display: cells stained with isotype controls (used to define fluorescence thresholds), unstimulated cells (Medium), cells stimulated with Pool 1–5, with PT-G, and with PHA used as a positive control.

**
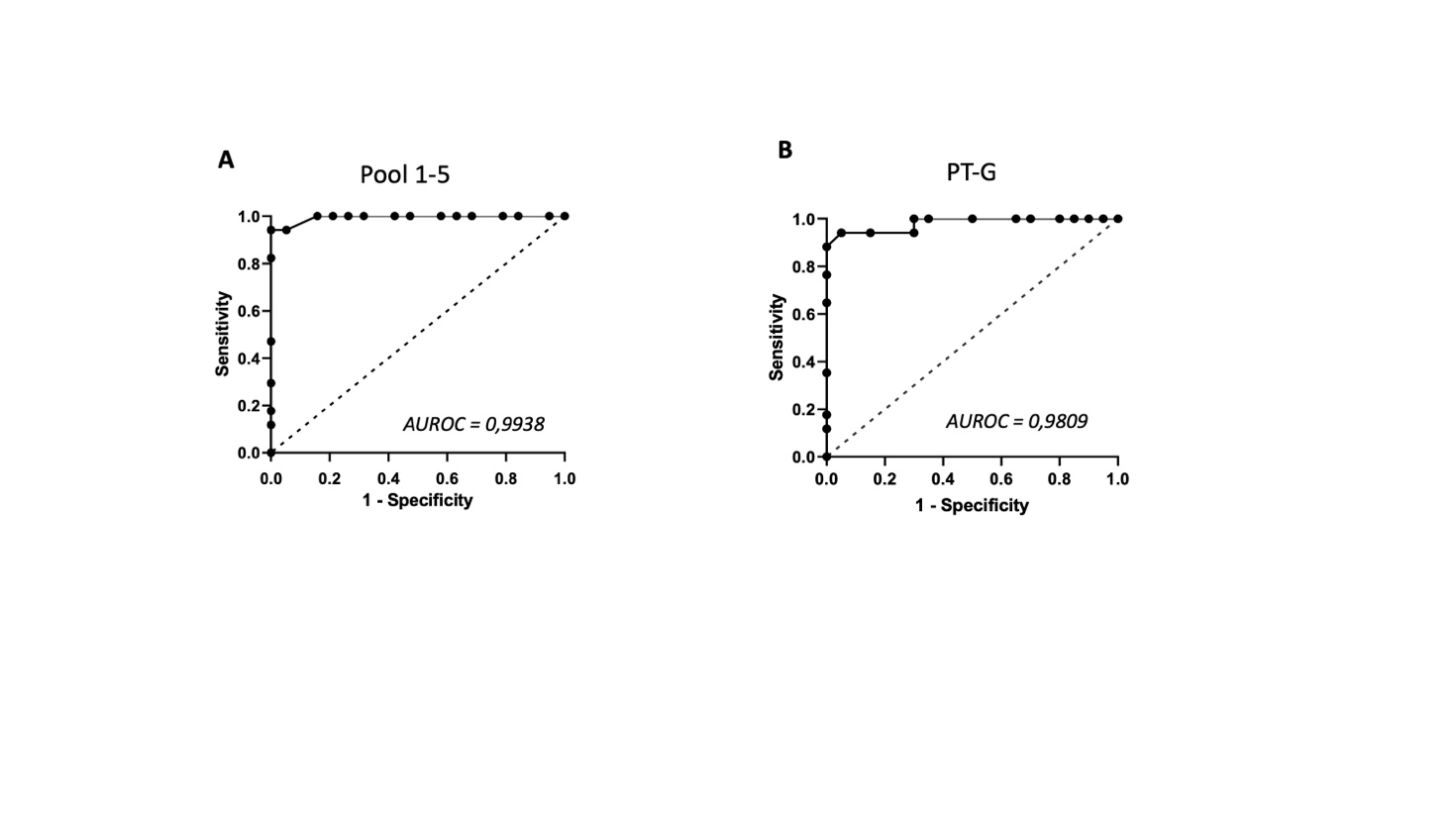
**

**Supplemetary Figure 3.** *ROC Analysis*

ROC analysis provided the optimal cut-offs for CD3^+^/CD4^+^/OX40^+^/4-1BB^+^ cells from untreated CD patients compared to GFD treated patients stimulated with Pool 1-5 (**Panel A**) and PT-G (**Panel B**).

**Supplementary Table 1.** Patients enrolled for G.A.T.CD4 analysis

**Supplementary Table 2.** Immunodominant gliadin peptides included in Pool 1-5
